# Correlation between nasopharyngeal carcinoma tumor volume and the 2002 International Union Against Cancer tumor classification system

**DOI:** 10.1186/1748-717X-8-87

**Published:** 2013-04-11

**Authors:** Zheng Wu, Mo-Fa Gu, Rui-Fang Zeng, Yong Su, Shao-Min Huang

**Affiliations:** 1 State Key Laboratory of Oncology in Southern China, Department of Radiation Oncology, Cancer Center, Sun Yat-sen University, Guangzhou, 510060, People’s Republic of China; 2 Department of Radiation Oncology, Tumor Hospital Xiangya School of Medicine of Central South University, Changsha, 410013, People’s Republic of China; 3 Department of Tumor, the First Affiliated Hospital, Sun Yat-sen University, Guangzhou, 510060, People’s Republic of China

**Keywords:** Nasopharyngeal carcinoma, Primary tumor volume, TNM classification, Intensity-modulated radiation therapy

## Abstract

**Background:**

The correlation between primary tumor volume and nasopharyngeal carcinoma (NPC) UICC 2002 T classification, N classification and distant metastasis after radiation therapy was discussed to provide further evidence for the inclusion of tumor volume into the TNM classification staging system.

**Methods:**

Between February 2001 and December 2008, 666 patients with NPC treated with intensity-modulated radiation therapy (IMRT) were analyzed retrospectively. Primary gross tumor volume was calculated from treatment planning computed tomography scans. The Kruskal-Wallis and Mann–Whitney tests were used for comparison of continuous variables and the chi-square test was used for categorical variables. A logistic regression model was used for multivariate analysis.

**Results:**

Median primary tumor volume of the 666 patients was 20.35 ml (range, 0.44 − 192.63 ml), and it gradually increased with T classification. Statistically significant differences in tumor volume were observed between patients with different T classifications (*p* < 0.001). The cervical lymph node metastasis rate was 64.7% (430/666); the differences in primary tumor volume between patients with or without lymph node metastasis were statistically significant (*p* < 0.001). Posttreatment distant metastasis occurred in 100 NPC patients, and the five-year distant metastasis-free survival was 84.2%. Univariate and multivariate analyses showed that N classification (*p* < 0.001) and tumor volume (*p* = 0.007) were the main factors influencing distant metastasis.

**Conclusion:**

Tumor volume was correlated with T classification, cervical lymph node mestastasis and distant metastasis after radiation therapy in nasopharyngeal carcinoma, suggesting that tumor volume should be included into the TNM staging system.

## Background

The TNM classification of malignant tumors is based on three components: the primary tumor, regional lymph node metastasis and distant metastasis [1]. In an ideal tumor staging system, the tumor (T) classification should not only reflect the status of local invasion, but also have close correlationships with regional lymph node metastasis and distant metastasis. However, the limitations of the current T staging system of nasopharyngeal carcinoma for determining tumor burden have been described in several studies [2,3]. The T classification system has also been shown to have a poor ability to predict distant metastases and overall survival in patients treated with intensity-modulated radiation therapy (IMRT) [4,5]. Therefore, it is important to establish new prognostic and staging indicators to improve the existing T classification system.

Tumor volume has a linear relationship with tumor burden and is considered the most direct indicator of tumor burden, which is significantly correlated with the prognosis of most of malignant tumors [6-8]. In addition, the interrelationship between tumor volume and radiation dose is a fundamental principle of modem radiation biology. The most direct explanation for these is that tumor volume is correlated with the number of clonogenic tumor cells to be eliminated. Moreover, the insufficient blood supply at the center of tumors with a large volume causes an increase in the number of hypoxic and quiescent cells, resulting in poor sensitivity of tumor cells to radiation and the need for a higher radiotherapy dose to achieve curative effects. Dubben et al. reviewed the effect of tumor volume on the outcome of radiotherapy in several malignancies and concluded that tumor volume is the most precise and most relevant predictor of radiotherapy outcome. Individual tumor volumes should therefore be included in clinical studies and considered in data analyses [8].

In nasopharyngeal carcinoma, tumor volume is not included in the UICC TNM staging system, Ho’s staging system or the Chinese 1992 staging system [1,9,10]. The T stagings of these classification systems are mainly based on local anatomic invasion, and the cranial nerve involved, and lack indicators that can objectively and quantitively reflect tumor burden. Several studies have demonstrated that the prognosis prediction for patients undergoing conventional radiotherapy or IMRT for nasopharyngeal carcinoma using tumor volume is superior than with the T staging system, and suggested that tumor volume should be included in the TNM staging of nasopharyngeal carcinoma [2,11,12]. However, the inclusion of tumor volume into the existing TNM staging framework requires further study, and the correlation between tumor volume and TNM staging also needs to be clarified. Although the correlation between tumor volume and the T classification has been investigated, few studies have addressed the correlation between the N classification and distant metastasis after radiotherapy. Therefore, the aim of the present study was to explore the correlation between tumor volume and the T and N classifications and distant metastasis after radiotherapy, and to provide further evidence for the inclusion of tumor volume into the TNM staging system.

## Methods

### Patients

Between February 2001 and December 2008, 1070 stage I-IVB NPC patients with definite histopathology diagnosis underwent IMRT at the Cancer Center of Sun Yat-Sen University. Of these patients, 404 cases were excluded from the study including 392 patients who received neoadjuvant chemotherapy and 12 patients who underwent neck mass resection biopsy before radiotherapy. A total of 666 patients who underwent pure radiotherapy, concurrent chemoradiotherapy, or concurrent chemoradiotherapy with adjuvant chemotherapy were included in the retrospective analysis.

The routine pretreatment evaluation included a complete medical history, physical examination, nasopharyngoscopy, chest radiography, abdominal region ultrasonography, bone scintigraphy, hematologic and biochemical profile, and magnetic resonance imaging (MRI) or computed tomography (CT) of the head and neck. All patients underwent disease staging using the UICC 2002 staging system [1].

### Patient Immobilization and CT Simulation

Patients were immobilized in a supine position with a head-neck-shoulder thermoplastic mask. CT simulation (Plus 4, Siemens, Erlangen, Germany) was performed at a slice thickness of 3 mm from the vertex of the head to 2 cm below the clavicle. Plain CT and contrast-enhanced CT images were obtained and transferred to the inverse IMRT workstation (CORVUS 3.0/3.2, Peacock plan) developed by the NOMOS Corporation.

### Target delineation and primary tumor volume measurement

The definition of target volumes and critical adjacent organs, the prescribed doses and plan evaluation criterion were described previously by Xiao *et al.*[13]*.* All target volumes (gross tumor volume and two clinical target volumes) and critical adjacent organs were outlined slice-by-slice on the axial fused images of plain and contrast-enhanced CT images in the treatment planning system in combination with MRI images. The treatment plans were evaluated and approved by three to four radiation oncologists specializing in NPC. The plan was approved upon meeting the evaluation criteria of our institution.

The system can automatically reconstruct a 3D image and calculate the volume of the tumor targets and critical organs. In this study, primary gross tumor volume (GTVp) included the gross tumor volume of the primary tumor and enlarged retropharyngeal lymph nodes.

### Treatment

Radiation was delivered with a linear accelerator (Varian, Palo Alto, CA) using 6 MV photons. IMRT was delivered with a dynamic multi-leaf intensity-modulating collimator (NOMOS Corporation, Sewickley, PA) using a slice-by-slice arc rotation approach. In our protocol, primary tumors and the upper neck above the cricoid cartilage were treated with IMRT, whereas the lower neck and the supraclavicular fossae were treated with a single anterior split field by conventional RT. The details were described previously by Xiao *et al.*[13]*.*

Of all cases included, 373 patients with stage II–IV disease received chemotherapy, 321 patients received concurrent chemotherapy, and 52 patients received concurrent chemotherapy plus adjuvant chemotherapy. All chemotherapy regimens were based on platinum drugs.

### Follow-up

Events were measured from the beginning of IMRT. After radiotherapy completion, the patients were subsequently followed-up monthly for the first 3 months. Then, patients were followed-up every 3 months from 3 months through 3 years, and every 1 year thereafter. Complete physical and fiberoptic nasopharyngoscopy or indirect nasopharyngeal speculum examinations were performed. Biochemistry profiles, chest radiography, abdominal ultrasonography, and MRI of the nasopharynx and cervical region were routine elements of the assessment. Further investigations were arranged as indicated.

### Statistical methods

The different groups were compared with respect to baseline characteristics, using the Kruskal-Wallis and Mann–Whitney tests for comparison of continuous variables and the chi-square test for categorical variables. A logistics regression model was used for multivatiate analysis. The Kaplan-Meier method was used for survival analysis. SPSS 13.0 software (SPSS Inc, Chicago, IL, USA) was used for all data analyses. Statistical significance was set at *p* ≤ 0.05.

## Results

### Patient characteristics

Patient characteristics are listed in Table 1. The median follow-up time was 51 months (range, 3–130 months) by December 31, 2011.

**Table 1:** Patient characteristics

**Characteristics**	**Number (%)**
**Sex**	
Male/Female	527/139 (79.1/20.9%)
**Age (years)**	
Median/Range	43/10-78
**Histology**	
WHO I/II/III	8/55/603 (1.2/8.3/90.5%)
**T classification**	
T1/T2/T3/T4	80/243/250/93 (12.0/36.5/37.5/14.0%)
**N classification**	
N0/N1/N2/N3	236/245/172/13 (35.4/36.8/25.8/2.0%)
**Clinical stage**	
I/II/III/IV	57/189/315/105 (8.5/28.4/47.3/15.8%)

*Abbreviations: WHO indicates World Health Organization*.

During follow-up, thirty-three cases of local failure were found, distant metastasis occurred in 100 cases, and 101 patients died. The five-year local failure-free survival, distant metastasis-free survival, and overall survival were 95.1%, 84.2% and 85.4%, respectively.

### Relationship between tumor volume and T classification

**Figure 1:**
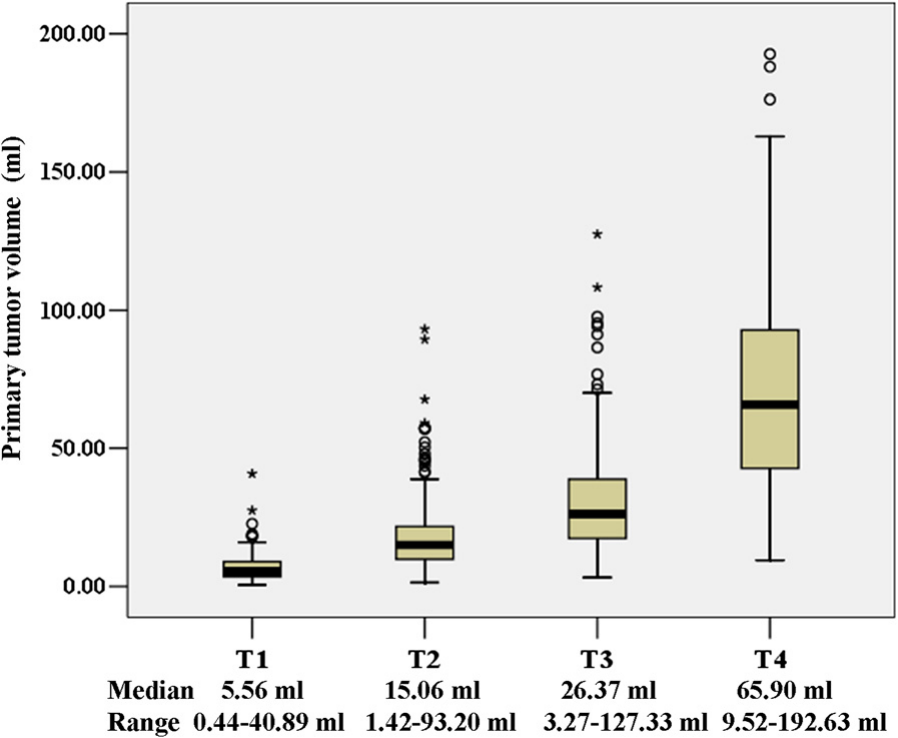
Correlation between T classification and primary gross tumor volume.

The median primary tumor volume of the 666 patients was 20.35 ml (range, 0.44 − 192.63 ml), and it gradually increased in correlation with T classification. The median primary tumor volume for the T1 classification was 5.56 ml (range, 0.44 − 40.89 ml); for the T2 classification, 15.06 ml (range, 1.42 − 93.20 ml); for the T3 classification, 26.37 ml (range, 3.27 − 127.44 ml); and for the T4 classification, 65.9 ml (range, 9.52 − 192.63 ml). The differences in tumor volume among the different T stages were statistically significant by the Kruskal-Wallis test (*p* < 0.001). The differences in primary tumor volume between the T2 and T1, T3 and T2, and T4 and T3 classifications were statistically significant (*p* < 0.001, all) according to the Wilcoxon test. The distribution of primary tumor volumes among different T stages is illustrated in Figure 1. A wide variation in tumor volumes was observed within the same T classification, in particular in advanced tumors. However, there was also overlap between the volumes in the different T classifications.

### Relationship between tumor volume and N classification

The cervical lymph node metastasis rate for nasopharyngeal carcinoma in the present study was 64.7% (430/666). The median nasopharyngeal tumor volume of N0 classification patients was 14.6 ml (range, 0.44 − 106.74 ml); in N + patients, the median tumor volume was 24.59 ml (range, 1.11 − 192.63 ml). The differences in primary tumor volume between patients with or without lymph node metastasis were statistically significant, as determined by the Mann–Whitney test (*p* < 0.001). In N + patients, the median nasopharyngeal tumor volume of N1-stage patients was 23.09 ml (range, 1.44 − 188.02 ml), that of N2 patients was 27.21 ml (range, 1.11 − 192.63 ml), and that of N3-stage patients was 23.03 ml (range, 10.81 − 53.87 ml). Statistical analysis showed that the differences in tumor volume among N1, N2 and N3 stages were not significant (*p* = 0.097).

### Relationship between tumor volume and distant metastasis

The median tumor volume in patients with distant metastasis was 35.75 ml (range, 5.22 − 192.63 ml), and that in distant metastasis-free cases was 18.25 ml (range, 0.44 − 188.02 ml). The differences in primary tumor volume between patients with or without distant metastasis were statistically significant by the Mann–Whitney test (*p* < 0.001). The chi-square test showed that T classification (*p* < 0.001) and N classification (*p* < 0.001) were significantly associated with distant metastasis. Further multivariate logistic analysis showed that N classification (*p* < 0.001) and tumor volume (*p* = 0.007) were the two main factors influencing distant metastasis after treatment, whereas T classification did not significantly correlate with distant metastasis (Table 2).

**Table 2:** Results of multivariate analysis: risk factors for distant metastasis in nasopharyngeal carcinoma

**Variables**	**Hazard ratio**	**95% confidence interval**	***p *value**
**Primary tumor volume**	1.012	1.003-1.022	0.007
**T classification** T1	1.0	ref	0.152
T2	6.291	0.827-47.863	
T3	8.690	1.146-65.888	
T4	7.488	0.882-63.574	
**N classification** N0	1.0	ref	<0.001
N1	2.009	1.018-3.962	
N2	4.095	2.080-8.063	
N3	11.931	3.454-41.220	

*Abbreviations: ref indicates reference*.

## Discussion

vThe results of the present study indicated that there is a close relationship between tumor volume and T classification. The Kruskal–Wallis test showed that tumor volume increased significantly with advancing T classification. Similarly, the Wilcoxon rank-sum test indicated that tumor volume was significantly greater in patients with more advanced T classifications than in those with adjacent early T classifications. However, there was significant variation within the same T classification, in particular in advanced tumors. At the same time, obvious overlaps were observed in the different T classifications (Figure 1). These results were consistent with those of Chua *et al.*[2] and Chong *et al.*[3], and provided further evidence that the existing T staging system is limited with respect to the assessment of tumor burden. Similar cases could be observed in clinical practice. For example, the invasion of the skull base in nasopharyngeal carcinoma is considered as a T3 classification, despite significant differences in the degree of invasion; single or multiple skull bases may be invaded, and even when the same skull base is invaded, the severity of the invasion can also differ. A study has confirmed that differences in the severity of skull base invasion are associated with different prognoses [14]. Therefore, the deficiency of the T staging in this respect may affect the formulation of a treatment strategy. This may cause deviations in the judgement of disease severity and the prognosis of patients. In addition, conservative treatment strategies adopted for large-volume tumors of an early stage may also result in ineffective therapy, even treatment failure. Therefore, tumor volume should be included in the T staging system to improve the determination of tumor burden, accurately identify the condition of the patient, and assist in the selection of a suitable treatment strategy.

Although the patterns of lymph node metastasis of nasopharyngeal carcinoma have been investigated [15,16], few studies have examined the relationship between nasopharyngeal tumor volume and cervical lymph node metastasis. In the present study, the lymph node metastasis rate was 65.4%. Statistical analyses showed that there were significant differences in nasopharyngeal tumor volume between patients with or without lymph node metastasis. The increase of primary tumor volume could have resulted in the infiltration of the parapharyngeal space and invasion of surrounding structures, which have been shown to have a close relationship with cervical lymph node metastasis [17,18]. However, in the present cases, the minimum tumor volume in patients with lymph node metastasis was only 1.11 ml. In the patients with lymph node metastasis, there were no significant differences in tumor volume in the different N classifications, which could be attributed to the high lymph node metastasis rate of nasopharyngeal carcinoma and metastasis occurrence in early-stage patients.

The majority of nasopharyngeal carcinoma cases are characterized by the presence of poorly differentiated or undifferentiated tumors, with high probability of distant metastasis. In patients undergoing IMRT, distant metastasis is also the most important reason for treatment failure and high mortality rates [4,19]. Therefore, the analysis of the risk factors for distant metastasis in nasopharyngeal carcinoma patients is important. Studies have shown a close relationship between N classification status and distant metastasis [4,5]. However, deficiencies in the detection of distant metastasis on the basis of the T classification system have been reported [4,5], suggesting that the modification of the existing T staging system is necessary to accurately assess the risk of distant metastasis. Tumor volume could serve as a potential independent factor. Large tumor volumes are indicative of the proliferation of tumor cells over a relatively long period of time or a high proliferation rate and malignancy of tumor cells, which are associated with distant metastasis. However, previous studies have mainly focused on the influence of tumor volume on local control and overall survival, with little investigation into the relationship between tumor volume and distant metastasis [11,12]. In our study, univariate and multivariate analyses showed that N classification and tumor volume were the main factors influencing distant metastasis of NPC, suggesting that tumor volume may be useful for predicting distant metastasis. The possible incorporation of tumor volume into the T staging system may result in a modified TNM staging system with an improved capacity to predict the occurrence of distant metastasis.

However, in NPCs treated by conventional radiotherapies, tumor volume has not been considered as an important clinical prognostic factor. This could be due to the common occurrence of infiltration in nasopharyngeal carcinoma, as well as the irregular shape of tumors and the difficulties in assessing tumor volume. To overcome this problem, several authors have developed semiautomated or automated systems to measure tumor volume [20]. With the advent of three-dimensional conformal radiotherapy technology, it is already possible to precisely measure the primary tumor volume. The three-dimensional volume of tumors and the corresponding exposure dose and coverage also become crucial factors in the formulation, optimization and assessment of clinical radiotherapy schemes. These factors provide strong support for the inclusion of tumor volume as an important prognostic factor in clinical staging.

The present retrospective study had certain limitations. A total of 392 patients receiving neoadjuvant chemotherapy were excluded, as the delineation of the GTV target was influenced by chemotherapy in some cases. Notably, the exclusion of patients receiving neoadjuvant chemotherapy would also affect an investigation into the effect of chemotherapy in advanced stage patients. At the same time, the patients who received induction chemotherapy were mainly III-IV stage patients. The exclusion of these patients from our study also affected the distribution of patients.

## Conclusions

The present study demonstrated that tumor volume was closely related with nasopharyngeal carcinoma T classification, cervical lymph node metastasis and distant metastasis after radiotherapy. The current T staging system has certain limitations in accurately reflecting tumor burden; variation within the same T classification was wide, especially for advanced tumors. Meanwhile, obvious overlaps were observed in the different T classifications. Tumor volume may be a potential indicator for predicting distant metastasis in nasopharyngeal carcinoma. Therefore, the inclusion of tumor volume into the UICC staging system is reasonable and feasible for nasopharyngeal carcinoma.

## Competing interests

The authors indicate no actual or potential conflicts of interest exist.

## Authors’ contributions

The authors contributions are the following: Zheng Wu and Mo-Fa Gu contributed with literature research, study design, data collection, data analysis, interpretation of findings and writing of the manuscript. Rui-Fang Zeng contributed with data collection and data analysis. Yong Su contributed with data collection, study design, critical review of data analyses, interpretation of findings and critical edit of the manuscript. Shao-Min Huang contributed with data collection. All authors read and approved the final manuscript.
